# Forecasting Tuberculosis Incidence in Iran Using Box-Jenkins Models

**DOI:** 10.5812/ircmj.11779

**Published:** 2014-05-05

**Authors:** Mahmood Moosazadeh, Mahshid Nasehi, Abbas Bahrampour, Narges Khanjani, Saeed Sharafi, Shanaz Ahmadi

**Affiliations:** 1 Department of Biostatistics and Epidemiology, Faculty of Health, Kerman University of Medical Sciences, Kerman, IR Iran; 2 Department of Epidemiology, School of Public Health, Iran University of Medical Sciences, Tehran, IR Iran; 3 Neurology Research Center, Shafa Hospital, Kerman University of Medical Sciences, Kerman, IR Iran; 4 Monash Centre for Occupational & Environmental Health, School of Public Health and Preventive Medicine, Monash University, Melbourne, Australia; 5 Center for Diseases Control and Prevention, Ministry of Health and Medical Education, Tehran, IR Iran

**Keywords:** Tuberculosis, Forecasting, Iran, Hb Jenkins

## Abstract

**Background:**

Predicting the incidence of tuberculosis (TB) plays an important role in planning health control strategies for the future, developing intervention programs and allocating resources.

**Objectives:**

The present longitudinal study estimated the incidence of tuberculosis in 2014 using Box-Jenkins methods.

**Materials and Methods:**

Monthly data of tuberculosis cases recorded in the surveillance system of Iran tuberculosis control program from 2005 till 2011 was used. Data was reviewed regarding normality, variance equality and stationary conditions. The parameters p, d and q and P, D and Q were determined, and different models were examined. Based on the lowest levels of AIC and BIC, the most suitable model was selected among the models whose overall adequacy was confirmed.

**Results:**

During 84 months, 63568 TB patients were recorded. The average was 756.8 (SD = 11.9) TB cases a month. SARIMA (0,1,1)(0,1,1)_12_ with the lowest level of AIC (12.78) was selected as the most adequate model for prediction. It was predicted that the total nationwide TB cases for 2014 will be about 16.75 per 100,000 people.

**Conclusions:**

Regarding the cyclic pattern of TB recorded cases, Box-Jenkins and SARIMA models are suitable for predicting its prevalence in future. Moreover, prediction results show an increasing trend of TB cases in Iran.

## 1. Background

Tuberculosis (TB) is an infectious disease caused by *Mycobacterium tuberculosis* and can affect different body organs (1, 2). TB disease can result from a rapidly progressive disease following recent infection with *Mycobacterium tuberculosis* or from reactivation of a past latent TB infection. Reactivation of disease is more common in countries that have controlled transmission properly, but recent transmission is more common in endemic countries (3-5). In Millennium Development Objectives which was agreed upon in September 2000 in the United Nations and which was accepted by 189 countries, the TB control program must achieve the objectives of reducing 50% of mortality from TB in comparison with 1990; stopping or reducing its incidence and prevalence until 2015 and finally eliminating (dropping its incidence to less than one case per million population) in 2050. Accordingly, the global plan to stop TB started its activity in January 2006 with an investment of more than 67 billion dollars and presented guidelines and strategies to control or eliminate tuberculosis based on dynamics of TB infection in societies (3, 6, 7). According to the World Health Organization (WHO) report, the rate of TB incidence has decreased from 142 cases per 100,000 people in 2004 to 139 cases in 2007 (3, 6). In Iran, considerable success has been achieved by integrating the TB control program in health and treatment network systems and by applying effective and organized management. TB incidence in Iran has decreased from 142 cases per 100,000 people in 1964 to 14.6 cases in 2011. In spite of these achievements and other effective attempts, achieving the predicted objectives is very difficult due to some uncontrollable problems including proximity to Pakistan and Afghanistan which are among the 22 highly infected countries of the world, proximity to Iraq (with its recent health crises) and to other newly independent countries in the north of Iran (with high prevalence of multidrug-resistant TB) (3, 6, 8).

Epidemiological studies have long been used to explain TB incidence and prevalence and its mortality. Considering epidemiological transition, emerging of Multi Drug Resistant (MDR) and Extensively Drug Resistant (XDR) TB and spread of HIV/AIDS, they can predict new challenges and present solutions (9, 10). Reviewing temporal changes and prediction of tuberculosis can have an important role in presenting health problems in the future; such as developing and expanding controlling and intervention programs and allocating resources optimally (11). To predict tuberculosis incidence and to study its temporal changes, different mathematical and statistical models have been used in different studies (12-16), and based on data nature and evaluation, a certain model has been used in every study. For example, Abdullah et al. applied a univariate time series model to the TB incidence data in Malaysia in order to determine the best forecasting model (13) or Kilicman et al. after appraisal of differential models showed that Holt’s trend corrected exponential smoothing is the best forecasting model, followed by the quadratic trend model (14). Zhang et al. in Chongqing used the ARIMA model in order to forecast tuberculosis (15). Also, regarding temporal changes of TB incidence, a variety of temporal patterns showing the peaks in spring and/or summer have been reported (11, 17-24).

## 2. Objectives

Due to the afore mentioned reasons, the millennium development objectives and the absence of such research in Iran, the present study was designed in order to predict the incidence of TB until 2014 using time series analysis and selection of a suitable model.

## 3. Materials and Methods

This was a longitudinal study. Tuberculosis data from April 2005 until April 2011 was obtained from the Ministry of Health and Medical Education of Iran, Center for Disease Control, Bureau of Tuberculosis.

The time unit used in this time series study was month. Daily records were aggregated into month and the series of monthly cases created 84 data points. In this study spring includes three months of April, May and June; summer includes July, August and September; autumn includes October, November and December; and January, February and March are the three months of winter. In order to compare the effect of season and month on registered cases, ANOVA was used. According to the results of the 2011 census, Iran has a population of 75,149,669 (25). In order to calculate the population of the studied years and to determine TB incidence per 100,000 the population of 2012 through 2014 was evaluated using the results of the census taken for 2006 and 2011 and considering the growth rate of 1.62% for the years 2005 to 2011 and 1.29% for the years 2012 to 2014.

### 3.1. Ethical Considerations

This study used the summed numbers of TB cases detected per month, and no identification or patients’ personal information was revealed.

### 3.2. Modeling Approach and Evaluation

Box Jenkins models forecast using moving averages, auto regression and a combination of these two. The steps in making Box Jenkins models includes; recognizing the pattern, fitting a model and forecasting. The ARIMA (Autoregressive Integrated Moving Average) models are a general class of models also known as Box-Jenkins models. The seasonal ARIMA model (SARIMA) is an expanded form of ARIMA, which allows for seasonal factors to be reflected (26, 27). In order to examine the nature of data, ts.plot time series graphs, ACF (autocorrelation function) and PACF (partial autocorrelation function) were initially depicted. Then the type of series was examined concerning stationary and non-stationary of the mean, variance and seasonal trend diagnosis. Also, Bartlett test was used to study the variance equality and Kolmogorov-Smirnov was used to study data normality. Since this series had a trend and non-stationary conditions in its average, one stage differencing was used to remove the trend and create stationary. In this study, the Box-Jenkins method was used to select the best fitted model. To make the model, an experimental model was first recognized from ARIMA models using real data analysis. The unknown parameters were estimated using ACF and PACF graphs before differencing, after differencing, without seasonal effect and with seasonal effect. Then according to the confidence interval of residuals, ACF and PACF graphs of the prepared model, meaningfulness of residuals of the prepared model through Ljong-Box tests, correlation coefficients of the real and experimental model, results of t-test for reviewing equality of the given parameter with zero and goodness of fit models, a suitable model was selected.

After examining different models, ultimately the univariate SARIMA model was used for forecasting. The SARIMA model consists of auto-regression, difference, and moving average, and is represented as SARIMA (p, d, q) (P, D, Q)s, in which (p, d, q) represents the non-seasonal part, (P, D, Q) represents the seasonal part and S represents the length of the seasonality. The p, d, q and P, D, Q represents the auto-regression, difference, and moving average, respectively (26). Statistical calculations were performed by Minitab 16. Different models can be obtained for various combinations of AR and MA individually and collectively. After the tentative model has been fit to the data, in order to test adequacy and performance of the constructed models, residual analysis was conducted and the Akaike Information Criterion (AIC), Bayesian Information Criterion (BIC), Ljung–Box–Pierce chi-square statistics and t-test for examination of null hypothesis of the parameters equalization to zero were calculated. Also correlation coefficient was used to exam the constructed model with actual data. The AIC and BIC values from the forecasting models were compared and the one with the smallest value was selected as the final forecasting model.

## 4. Results

**Figure 1.**
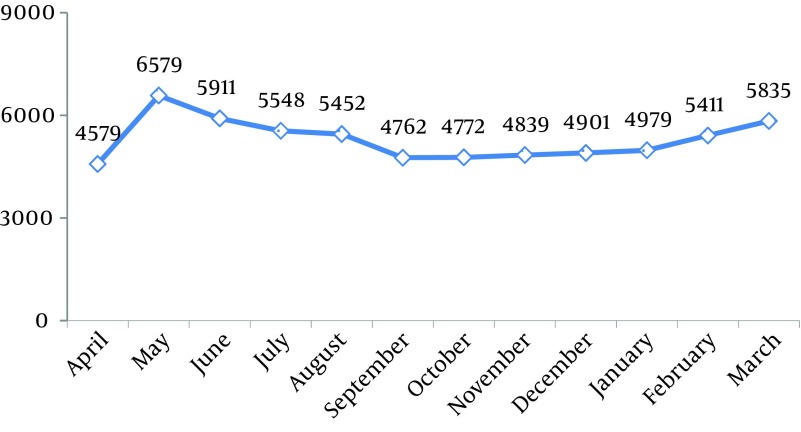
Aggeregated Monthly Number of Tuberculosis Total Incidence in Iran from 2005 Untill 2011

**Figure 2.**
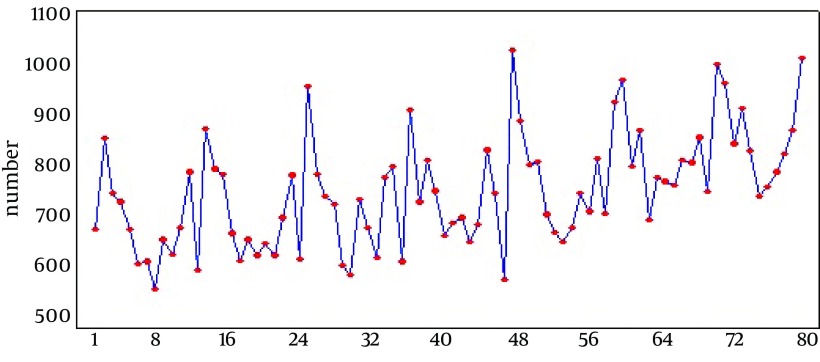
Time Series Plot of (Crud Data) T B in Cidence Number per Month from 2005 Until 2011

During this study (2005-2011), 63568 tuberculosis cases were recorded. The average recorded cases were 756.8 TB cases in a month with a standard deviation of 11.9. Among these 84 months, the minimum and maximum incidence was 566 and 1033 cases respectively. Bartlett’s Test revealed that the variances in this time series were equal (P value = 0.8). Also, according to the results of the Kolmogorov-Smirnov test, the distribution of this time series had the prerequisite of normality (KS = 0.07, P > 0.15). Based on Figure 1 and total monthly TB cases in 2005-2011, maximum and minimum cases were recorded in May (6579) and April respectively. ANOVA test, which was used to study differences between months and seasons of incidence, showed that TB cases were significantly more in May (with mean ± standard deviation of 939.9 ± 63.6) than in other months (F = 8.9, P < 0.001). Moreover, TB cases in spring with monthly mean and standard deviation of 812.8 and 142.2 were more than those in other seasons (F = 5.3, P = 0.002). Time series graph of raw data (Figure 2) shows that TB incidence has a seasonal pattern and this curve has a trend whose mean is non-stationary. This time series reached the stationary conditions required for fitness of Box-Jenkins after one differencing step on the original data and one seasonal differencing step. Therefore the amount of 1 was considered for the parameters d and D. According to the ACF graph, the parameters q and Q were more likely to be 1 too. Determining the level of p and P was uncertain according to the PACF graph and the lines were tangent on the zero assumption line. Therefore different levels were tested. Autoregressive (AR) and Moving Average (MA) approaches were examined separately; analysis graph of residuals (ACF and PACF) were significant and pure AR and MA approaches weren’t suitable for data. Various levels of non-seasonal ARIMA were also examined concerning various levels of p, d and q and according to model evaluation indexes, none of them was suitable. Thus, adding seasonal parameters "P, D and Q" was strongly suggested.

**Figure 3.**
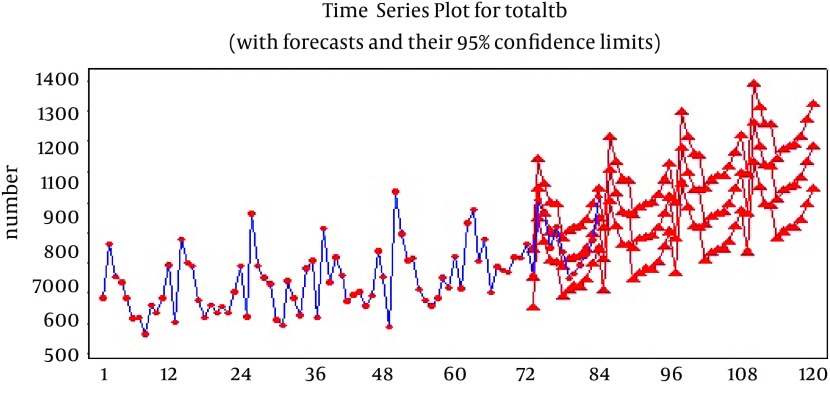
Observed and Predicted Number of Tuberculosis in Iran, Per Month From 2005 Until 2014

Table 1 shows 4 SARIMA models with a combination of MA and AR. The overall function of these four models was suitable based on graph of residuals (ACF and PACF) and Ljung-Box. The correlation coefficient was significant between real data and predicted data. However, the t-test which examined the equality of parameters with zero showed that AR was not significant in most added models and thus considering level "0" for non-seasonal (p) and seasonal (P) parameters was thought to have a better performance. The AIC and BIC were calculated for four models and their levels have been mentioned in Table 2. BIC levels were close for all four models and the best model with suitable parameters was selected based on AIC. Among these four models, SARIMA (0,1,1)(0,1,1)_12_ with the lowest AIC level (12.785) was selected as the most adequate and qualified model for prediction. Using this selected model SARIMA (0,1,1)(0,1,1)_12_, prediction was made for next 36 months (2012-2014) and the results are shown in Figure 3. Months 1 to 84 are based on real data, while months 85 to 120 are based on the predicted data. This graph also shows that confidence interval of the predicted levels is narrow which shows high adequacy of this model for prediction.

**Table 1.** Tests to Compare the Adequacy and Performance of the Constructed Model Type ^a^

Model Type	t-test (Parameters Equalization Values With Zero)			Residuals Plot		Ljung-Box (lag 12)		Correlation Coefficient (Model and Actual Data)	
	Lag	t	P	ACF	PACF	Chi-Square	P	r	P
**SARIMA (1,1,1)(1,1,1)_12_**				not significant	not significant	7.6	0.4	0.86	< 0.001
AR	1	-2.1	0.04						
SAR	12	-0.8	0.4						
MA	1	10.6	0.0001						
SMA	12	4.2	0.0001						
**SARIMA (2,1,1)(1,1,1)_12_**				not significant	not significant	5.4	0.5	0.87	< 0.001
AR	1	-2.2	0.03						
AR	2	-1.5	0.1						
SAR	12	-0.8	0.4						
MA	1	12.8	0.0001						
SMA	12	4.2	0.0001						
**SARIMA (0,1,1)(0,1,1)_12_**				not significant	not significant	12.3	0.2	0.86	< 0.001
MA	1	8.6	0.0001						
SMA	12	6.03	0.0001						
**SARIMA (2,1,1)(2,1,1)_12_**				not significant	not significant	6.7	0.2	0.84	< 0.001
AR	1	-2.3	0.03						
AR	2	-1.6	0.1						
SAR	12	-0.8	0.4						
SAR	24	-0.9	0.4						
MA	1	1260.3	0.0001						
AMA	12	0.9	0.3						

^a^ Abbreviations: ACF; autocorrelation function, AR; autoregressive, MA; moving average, PACF; partial autocorrelation function, SARIMA; seasonal autoregressive integrated moving average.

**Table 2.** Goodness of Fits for Models ^a^

Model Type	AIC	BIC
**SARIMA (0,1,1)(0,1,1)**_**12**_	12.785	7.785
**SARIMA (1,1,1)(1,1,1)**_**12**_	16.568	7.568
**SARIMA (2,1,1)(1,1,1)**_**12**_	18.542	7.542
**SARIMA(2,1,1)(2,1,1)**_**12**_	20.547	7.546

^a^ Abbreviation: SARIMA; seasonal autoregressive integrated moving average.

**Figure 4.**
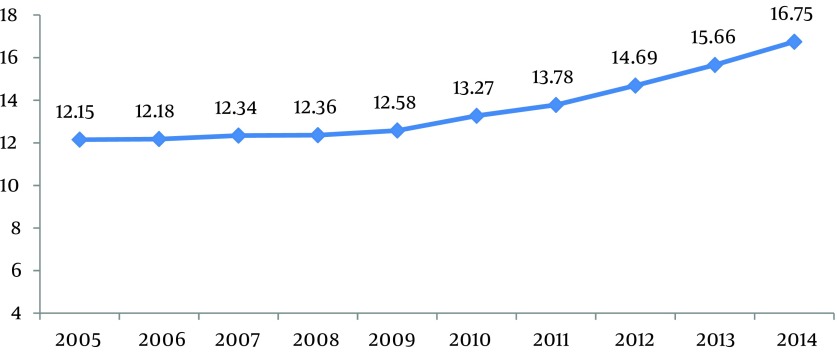
Incidence of Tb Total from 2005 Until 2014 Per 100000 Habitants

Based on real data and predictions, TB incidence was calculated from 2005 to 2014 per 100,000 people. Its incidence from 2005 to 2011 was determined according to real data, while its incidence for years 2012-2014 was based on prediction. Based on real data, TB total incidence varied from 12.15 in 2005 to 13.78 in 2011; its incidence for 2014 was predicted to be 16.75 (Figure 4).

## 5. Discussion

Our study revealed that the best prediction is possible with SARIMA (0, 1, 1)(0, 1, 1)12. TB prevalence has had an increasing trend from 2005 but this trend has risen more steeply from 2009. The overall TB incidence in 2014 is predicted to be 1.38 times the year 2005. The TB peak in Iran is in spring and in May. Among the different suggested models SARIMA (0,1,1)(0,1,1)_12_ was selected as the best fit for predicting TB prevalence. Zhang et al. used the ARIMA model to fit the changes of the incidence of tuberculosis in parts of China and to predict the incidence in future, which can be used as a scientific basis for the prevention and treatment of tuberculosis in this country (15). Permanasari et al. investigated the performance of six different forecasting methods, including linear regression, moving average, decomposition, Holt-Winter’s, ARIMA, and artificial neural network (ANN) for monthly tuberculosis data forecasting and showed that the ARIMA model was the most appropriate model (28). Na et al. in a study showed that the ARIMA model can be used to appropriately fit the changes of the incidence of pulmonary tuberculosis in Sichuan province of China and for short-term predictions (29). In their study, ARIMA (1,0,0)(1,1,0)_12_ exactly fitted the series of the previous months’ incidence. Xing et al. by comparison of three models of time series prediction including Grey System, Holt's linear exponential smoothing and ARIMA on prediction of incidence of pulmonary tuberculosis reported that the ARIMA model was the best choice (30). Other prediction studies with non-time series methods on tuberculosis data have been conducted as well, such as a study in Spain (18) with mathematical modeling on the registered cases of tuberculosis from 1971 until 1996 which has predicted the pattern for tuberculosis incidence and showed increases in the incidence. Another forecasting study on Malaysian tuberculosis data showed that the Double Exponential Smoothing technique was the best time series model comparing to Single Exponential Smoothing, Holt's Method and Holt Winter's Trend and Seasonality for both the multiplicative and additive effect assumption (13). Kilicman et al. applied the linear trend, quadratic trend, simple moving average, simple exponential smoothing and Holt’s trend corrected exponential smoothing for forecasting tuberculosis incidence in the Terengganu area in Malaysia. They showed that Holt’s trend corrected exponential smoothing is the best forecasting model, followed by the quadratic trend model. The results also showed that TB cases from 2009 until 2013 will increase (14).

Results of the present study match other studies about the selection of a suitable model which predict increases in TB cases. Considering the cyclic pattern of TB, time series analysis models are probably the most suitable to examine the trend and to predict TB incidence. As mentioned in the results of other studies, seasonal ARIMA model was more efficient than other time series analysis models in terms of predicting TB; e.g. , one of the its principal advantage is its capability to easily remove irregular changes and seasonal factors. The seasonal ARIMA model or SARIMA model is an expanded form of ARIMA, which allows for seasonal factors to be reflected (26). Despite the presence of an appropriate TB Surveillance and Control Program, the probable causes of increased TB in Iran can be attributed to increased HIV/AIDS, MDR-TB and immune debilitating diseases (Diabetes, cancers, etc.). According to our results, the peak of TB prevalence in Iran is in spring and May. Among the research done in other countries, the peak of tuberculosis in England (17) and Hong Kong (20) was summer, in America (24) was spring and autumn and in Taiwan (22) and Ireland (23) was spring and summer. Also in the north of India (19) the peak of tuberculosis was spring and summer, but a seasonal pattern was not seen in the south of India (19). However, in most studies the peak was seen in spring and summer which was similar to our study. There are hypotheses about the reason for the seasonal pattern of tuberculosis. One of the hypotheses is that due to cold weather, and confined spaces transmission happens in winter and eventually after passing the latency period, the presentation of symptoms and diagnosis of the disease happens in spring (31). Others think that acquiring tuberculosis in winter and spring is due to recurrent respiratory infection, influenza, immunodeficiency and also living in confined spaces, and the diagnosis of tuberculosis happens in spring (18) and other authors in Hong Kong showed that decrease in sunlight and decrease in vitamin D significantly increases the incidence of tuberculosis patients with positive smear and sputum culture (20).

Absence of multivariable models to predict TB and the uncertain status of most patients regarding HIV/AIDS and MDR-TB, especially in the first years of this study are some of the limitations of this research. The strength of our study was the fact that we used valuable data from the national tuberculosis surveillance program and we made predictions for the next few years. This information can be used for TB control programs. It is recommended that prevalence of HIV/AIDS, MDR-TB and diabetes in tuberculosis patient be estimated in Iran using statistical modeling; then, the reasons of increased TB cases can be justified using their results as independent variables in modeling. Considering the effects of uncontrollable factors (such as the high prevalence of TB in neighboring countries) and millennium development objectives (which aims to stop or decrease the trend of TB by 2015); reviewing strategies and guidelines, strengthening human and financial resources and supporting the TB Control Program are of great importance.

Due to the cyclic pattern of tuberculosis incidence, Box-Jenkins SARIMA models are suitable for prediction. The results of this prediction show an increasing trend in total TB incidence in Iran.
